# Sarcopenia and Pleural Mesothelioma: The Current Knowledge

**DOI:** 10.3390/muscles3010006

**Published:** 2024-02-08

**Authors:** Nikolaos D. Karakousis, Konstantinos I. Gourgoulianis, Nikolaos Papanas, Ourania S. Kotsiou

**Affiliations:** 1 Department of Respiratory Medicine, Faculty of Medicine, University of Thessaly, 41110 Larissa, Greece; kgourg@uth.gr (K.I.G.); okotsiou@uth.gr (O.S.K.); 2 Second Department of Internal Medicine, Diabetes Centre-Diabetic Foot Clinic, Democritus University of Thrace, 68100 Alexandroupolis, Greece; papanasnikos@yahoo.gr; 3 Laboratory of Human Pathophysiology, Faculty of Nursing, University of Thessaly, 41500 Larissa, Greece

**Keywords:** sarcopenia, low muscle mass, low muscle strength, pleural mesothelioma, lung disease

## Abstract

Pleural mesothelioma (PM) is a tumor related to adverse prognosis. The PM WHO classification has mainly identified three major subtypes of PM which are epithelioid, biphasic, and sarcomatoid. Sarcopenia is a medical issue related to a reduction in muscle mass and strength. It represents a major health issue globally because it is related to adverse effects such as hospitalization, increased length of stay, disability, increased morbidity and mortality and augmented health care expenditures. In this literature review, we attempted to examine the upcoming association between sarcopenia and PM. As recorded by the current literature, muscle loss in PM subjects was related to poorer survival and lower levels of activity. Subjects with PM had increased rates of pre-sarcopenia and malnutrition, while pre-sarcopenia was related to worse activity levels, and malnutrition was related to worse quality of life (QoL). Both tumor volume and sarcopenia were related to long-term mortality in surgically treated PM subjects, while sarcopenia was present both pre-operatively and post-operatively in these subjects. In addition, post-operative sarcopenic subjects showed a decreased 3-year overall survival (OS) in comparison with those who did not have sarcopenia, while pre-operative sarcopenia was importantly related to an increased rate of post-operative adverse outcomes. More studies are needed to validate these claims.

## 1. Introduction

Mesothelial tumors are categorized into preinvasive or benign tumors and mesotheliomas [1]. The preinvasive or benign tumors consist of well-differentiated papillary mesothelial tumors, adenomatoid tumors and mesothelioma in situ, while malignant tumors are mesotheliomas. Malignant tumors might be diffuse or localized [1]. Diffuse mesothelioma is an infrequent malignancy deriving from mesothelial cells which line the pleural and peritoneal cavities along with the tunica vaginalis testis and pericardium [2].

Pleural mesothelioma (PM) is a tumor related to adverse prognosis. Over the past decade, PM incidence has increased firmly worldwide, while an estimation associated with 2008 data suggested an average of 14,200 cases globally every year [3]. The PM WHO classification has mainly identified three major subtypes of PM which are epithelioid, biphasic, and sarcomatoid [1,2,4]. Concerning symptoms, most of the subjects with PM might present breathlessness, chest pain or both, and making a diagnosis, via methods including radiological imaging and the sampling of pleural fluid for biochemical and cytological investigation, is quite challenging [3]. PM management might include options such as chemotherapy, targeted therapy and radiotherapy, while the surgical approach benefit in PM is much debated [3]. According to the SEER database, the median survival in subjects diagnosed with epithelioid, biphasic, and sarcomatoid PM after surgical management is 19, 12, and 4 months, respectively [1].

Epithelioid mesothelioma (EM) is related to approximately 80% of all PMs and includes epithelioid (rounded/polygonal) rather than spindle-shaped cells [2,4]. EM consists of deceptively bland, uniform cuboidal cells. These cells penetrate the pleura in a tubulo-papillary growth pattern, formed by round-to-oval structures admixed with tumor cells which cover a fibrovascular core [2,4]. Mesothelioma named as sarcomatoid is recorded as the second most repeated subtype of PM and has been related to only 4-month survival in subjects after surgical confrontation [2,4]. The WHO classification describes it as a proliferation of spindle cells arranged in fascicles or in haphazard patterns penetrating the lung parenchyma or adipose tissue. Necrosis and atypical mitoses might be also attending [2,4]. Biphasic mesotheliomas consist of epithelioid and sarcomatoid morphologies at the same time, and at least 10% of every one component is essential for the final diagnosis concerning resection specimens (extrapleural pneumonectomy/extended pleural decortication). The diagnosis of biphasic mesothelioma can be carried out in small biopsies [2].

It is already established that epithelioid PM is related to greater prognosis in comparison to biphasic and sarcomatoid subtypes [4]. The final confirmation of diffuse malignant PM relies on the pathologic evaluation of tumor tissue deriving from pleurectomy, core biopsy sampling or other more considerable resections [5]. Clinical manifestations might not be conclusive, and according to the extent of tumor participation, they may include dyspnea, night sweats, pleuritic chest pain and weight loss [5]. Concerning the pathogenesis of diffuse malignant PM, previous asbestos exposure was recorded in almost 70% of subjects. Other elements might concern therapeutic radiation exposure for prior malignancy, exposure to non-asbestos mineral fibers and chronic inflammatory issues [5]. In addition, germline variations in BRCA1-associated protein 1 (BAP1) and other tumor suppressors have been related to the evolution of diffuse malignant PM in a subset of subjects [5].

As for the treatment strategy, it is already well established that subjects with malignant PM might be treated with trimodality therapy including surgery, chemotherapy, and radiation therapy (RT) [5]. Two fundamental surgical techniques for malignant PM consist of extrapleural pneumonectomy (EPP), where the lung is removed en bloc, and pleurectomy/decortication, where the lung remains in situ [6]. Chemotherapy is often platinum-based, including cisplatin, usually in combination with a folate antimetabolite (for example, pemetrexed) [6].

Sarcopenia is a clinical issue which is related to a reduction in muscle mass and strength [7,8,9,10,11]. It represents a major health issue globally due to fact that it is related to unfortunate outcomes such as hospitalization, augmented length of stay, disability, increased morbidity and mortality and augmented health care expenditures [7,12,13]. It represents a progressive wastage of skeletal muscle mass and function and can be present not only in the elderly, but also in other chronic clinical conditions [12,14,15]. Moreover, sarcopenia is associated with the syndrome of frailty which is related to augmented age and chronic conditions [16,17,18,19]. The syndrome of frailty is characterized by decreased and/or incomplete recovery from various damaging elements such as injury, infection, surgery or psychosocial distress [20]. As a result, it seems essential to diagnose upcoming sarcopenia in general populations and try to manage and prevent its adverse outcomes. It must be distinguished from cachexia which is a systemic condition of wasting and basically considered a late-stage demonstration of long-standing diseases, such as malignancies, organ collapse, or infections [21].

In 2010, the European Working Group on Sarcopenia in Older People (EWGSOP) recorded a preliminary sarcopenia clarification, but in early 2018, the Working Group (EWGSOP2) tried to enrich the original definition aiming to include all the advances concerning sarcopenia that took place over the last decade [22]. Particularly, in its latest operational clarification, EWGSOP2 utilizes low muscle strength as the first element of sarcopenia, since muscle strength is currently the most trustworthy means of muscle operation, where sarcopenia is considered to be a potential condition when low muscle strength is present [22]. The diagnosis of sarcopenia is validated by the diagnosis of low muscle quantity or quality, but it is significant to mention that when low levels of muscle strength, muscle quantity/quality and physical performance are all present at the same time, the state of sarcopenia is considered severe [22].

Different tests are currently useful to define sarcopenia in everyday practice and scientific research, while the specific tool selection might me associated with the subject’s movability, the approach to technical resources in the specific healthcare test setting including community settings, the research center or hospital and finally the reason for testing which might include monitoring or rehabilitation and recovery [22].

One important means to find sarcopenic subjects is the utilization of the “Strength, Assistance with walking, Rising from a chair, Climbing stairs, and Falls” (SARC-F) questionnaire [22,23,24,25,26,27]. The SARC-F assessment is a self-reported questionnaire by subjects who are examined as a screening test for sarcopenia hazard assessments. Responses are related to the subject’s discrimination of his or her restrictions in strength, walking skill, rising from a seated position, stair climbing and previous falls [22,23]. The suggested cutoff value concerning SARC-F is ≥4 points [28]. The SARC-F assessment has been demonstrated to have good correlation with clinical outcomes in the elderly and an amount of underlying diseases, but it is also recorded that the SARC-F assessment has its imperfections including low sensitivity concerning sarcopenia [28].

Muscle strength can be assessed by measuring mainly grip strength, as it is recorded that low grip strength is an important forecaster of poor results such as longer days of hospitalization, augmented operational limitations, deteriorated quality of life (QoL), and increased mortality [22,29]. It is already well established that the evaluation of hand grip strength demands the utilization of a handheld dynamometer operating under well-controlled test conditions [22,29,30,31,32,33]. The hand grip strength (HGS) assessment is less expensive, does not demand complex training and can directly reflect the current muscle strength [34]. Nevertheless, currently, there is a great number of various methods of evaluating hand grip strength which makes the comparison among studies quite intriguing [35]. In addition, the utilization of the chair stand test that is also known as the chair rise test might be utilized as a means for evaluating the muscle strength of the legs [22,32].

Muscle quantity or mass can be assessed via many techniques, among them magnetic resonance imaging (MRI) and computed tomography (CT) which are the top techniques for non-invasive evaluations of muscle quantity or mass, even though they are quite expensive and have specific limitations concerning portability and the need of highly trained personnel [22,36,37,38,39]. Nevertheless, the most widely utilized means to assess muscle quantity (total body lean tissue mass or appendicular skeletal muscle mass) non-invasively is dual-energy X-ray absorptiometry (DXA) [22,40,41,42]. DXA is known to be the gold-standard means concerning the investigation of body composition at the molecular level, granting the evaluation and quantification of lean mass, fat mass and bone mineral content, both in a single body region of interest and at the whole-body level [43]. DXA is low-priced in comparison with a standard CT scan, and it is not difficult to be carried out technically. Nevertheless, DXA might have several restrictions [44]. Among them are low accuracy in estimating truncal fat and muscle because of the incapability to separate intra-abdominal organs, over-/underestimation of the extent of sarcopenia or the presence of obesity from the amount of fat and muscle interpolated from arms and legs and low accuracy when edema and altered hydration conditions are present [44]. In addition, the absence of demographics reference data and un-experienced image examination are frequent conditions that could reduce DXA effectiveness in everyday practice with potential implications for the correct classification of diagnosis and handling of subjects [44]. Another means of sarcopenia assessment is bioelectrical impedance analysis (BIA) which does not directly assess muscle mass but, on the contrary, provides an approximate of muscle mass based upon whole-body electrical conductivity [22,45,46,47].

As for a physical performance evaluation, it might be assessed by different kind of tests such as the gait speed test, the short physical performance battery (SPPB) test, and the timed-up and go (TUG) tests [22,48,49,50,51,52,53]. It seems that gait speed is a fast, highly reliable and safe means for assessing sarcopenia, and it is broadly utilized in everyday clinical application [22,32].

Other alternative and new tests that might assess muscle mass may include lumbar third vertebra imaging via CT, mid-thigh muscle measurements, psoas muscle measurements with CT, a creatine dilution test, and an ultrasound (U/S) assessment of the muscle. The validation of specific biomarkers indicative of sarcopenia diagnosis and monitoring seems to be an intriguing issue for the scientific community and a future challenge [22].

Regarding interventions to confront sarcopenia, resistance exercise (RE) is recommended currently as the best therapy for confronting the unfavorable results of sarcopenia [54,55,56,57]. Moreover, increasing protein and calorie intake, with protein additions if appropriate, is already recorded concerning dietary interventions [10,58,59]. Nevertheless, currently, it seems that there is no consensus concerning the optimum means of intervention on sarcopenic subjects.

In this literature review article, we examined the probable and upcoming association between sarcopenia and skeletal muscle disorder and pleural mesothelioma. It is already known that sarcopenia and cancer might be present simultaneously and have an impact on each other, while in lung cancer subjects, sarcopenia might be related to lung cancer prognosis through different mechanisms including oxidative stress and inflammation, even though more studies are required [60,61]. These data paved our way to study the interplay between pleural mesothelioma and sarcopenia.

## 2. Materials and Methods

**Figure 1:**
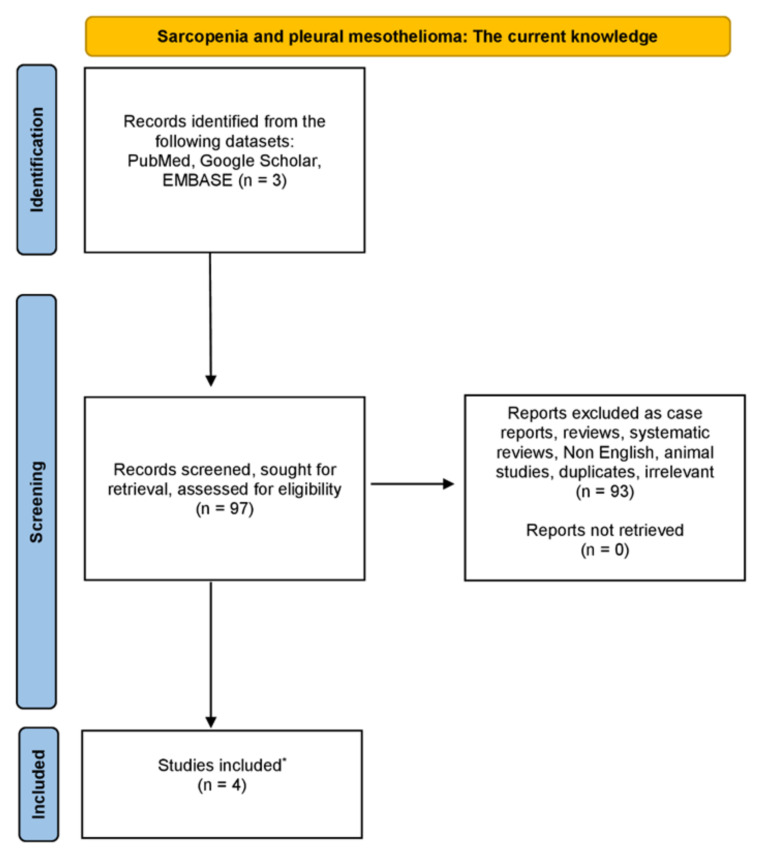
Diagram showing the literature review organization (* only original, written in English, non-animal studies were incorporated in this non-systematic review).

We have carried out a thorough examination in the databases of PubMed, Google Scholar and EMBASE, from August 1975 until December 2023, using combinations of the following keywords: “sarcopenia” OR “low muscle mass” OR “muscle mass” AND “pleural mesothelioma” OR “mesothelioma”. Only original studies written in English were incorporated in this non-systematic review article. Moreover, all the references of studies included were also rigorously investigated. Studies related to animals were excluded. The organization of the literature review is encapsulated in the flowchart diagram (Figure 1).

## 3. Results

The basic point of this non-systematic literature review article was to show the possibility of any upcoming interplay between sarcopenia and pleural mesothelioma, as recorded by the current literature. The results are presented in Table 1.

Jeffery et al. studied and determined the prevalence of pre-sarcopenia and malnutrition in malignant pleural mesothelioma (MPM) and investigated whether there was any difference in activity levels and QoL in accordance with nutritional conditions and body composition [62]. Subjects with an MPM diagnosis were recruited. Pre-sarcopenia was characterized as low appendicular skeletal muscle mass (ASM) (≤7.26 kg/m^2^ for men and ≤5.45 kg/m^2^ for women), measured via DXA [62]. Malnutrition was characterized as a rating of B or C on the Patient-Generated Subjective Global Assessment, and study results included objective activity levels (Actigraph GT3X) and health-related quality of life (HRQoL; Functional Assessment of Cancer Therapy General) [62]. Furthermore, 61 subjects participated in their study where 79% were males with a median age 69 (IQR 62-74) years and a median BMI of 25.8 (IQR 24.3–28.4) kg/m^2^. Moreover, 54% were pre-sarcopenic and 38% were malnourished [62]. Interestingly, the amount of time that was spent in light activity per day was decreased in subjects with pre-sarcopenia in comparison to subjects without sarcopenia [median 25.4 (IQR 19.8–32.1)% vs. 32.3 (27.1–35.6)%; *p* = 0.008] [62]. Subjects with malnutrition had worse HRQoL than well-nourished subjects [mean 69.0 (16.3) vs. 84.4 (13.3); *p* < 0.001] [62]. As a result, they concluded that MPM subjects had more pre-sarcopenia and malnutrition, while pre-sarcopenia was related to worse activity, and malnutrition was related to worse QoL [62].

Jeffery et al., conducting an observational study, examined in MPM subjects potential alterations in body composition and its association with activity levels, diet and survival [63]. This investigation was a secondary data analysis deriving from a longitudinal observational study of MPM subjects. Subjects included in this study completed 3-month evaluations for up to 18 months, and subjects with two DXA scans were included [63]. Alterations in ASM and total fat mass were utilized to categorize MPM subjects into phenotypes, while activity levels were assessed with an ActiGraph GT3X+ accelerometer. Energy and protein intake was assessed with a 3-day food record and 24 h recall [63]. Moreover, 18 subjects (89% men) in total were included in this study with a mean age of 68.9 ± 7.1 years [63]. The median period between DXA was 91 (84–118) days. In comparison with subjects with ASM maintenance (n = 9), fewer participants with ASM loss (n = 9) survived ≥12 months from follow-up (*p* = 0.002) [63]. It was also demonstrated that subjects with ASM loss had an augmented sedentary time (*p* = 0.028) and lowered light activity (*p* = 0.028) and step count (*p* = 0.008) [63]. Nevertheless, activity levels did not alter in subjects with ASM maintenance (*p* > 0.05), while both energy and protein intake did not demonstrated any alteration in either group (*p* > 0.05) [63]. They concluded that muscle loss was related to worse survival and lower levels of activity [63].

Verhoek et al. investigated the prognostic importance of sarcopenia, low precardial adipose tissue (PAT), and high tumor volumes in the outcome of surgically managed PM [64]. They conducted a retrospective study from 2005 to 2020 in which consecutive surgically managed PM subjects with a pre-operative CT scan were enrolled in this investigation [64]. Sarcopenia was evaluated via CT-based parameters assessed at the level of the fifth thoracic vertebra (TH5), excluding fatty infiltration based on CT attenuation. In addition, the outcomes were stratified for gender, and a threshold of the 33rd percentile was utilized to characterize sarcopenia [64]. On the other hand, both tumor volume and PAT were evaluated, while the outcomes were correlated with long-term mortality and progression-free survival [64]. In total, two hundred and seventy-eight PM subjects (252 male; 70.2 ± 9 years) were included. The mean progression-free survival was 18.6 ± 12.2 months, and the mean survival time was 23.3 ± 24 months [64]. Progression was related to chronic obstructive pulmonary disease (COPD) (*p* < 0.001), the type of surgery (*p* = 0.026) and the tumor stage (*p* = 0.001). Three-year mortality was related to increased subject age (*p* = 0.005), increased tumor stage (*p* = 0.015), the presence of COPD (*p* < 0.001), and increased tumor volume (*p* < 0.001) [64]. Kaplan–Meier statistics demonstrated that subjects with sarcopenia had an increased three-year mortality (*p* = 0.002). Even though there was an inverse correlation of progression-free survival and mortality with tumor volume (r = 0.281, *p* = 0.001 and r = −0.240, *p* < 0.001, respectively), a correlation with PAT was solely demonstrated for epithelioid PM (*p* = 0.040) [64]. As a result, the main outcomes of this study demonstrated that both sarcopenia and tumor volume are related to long-term mortality in surgically treated PM subjects, and even though there was an inverse correlation of progression-free survival and mortality with tumor volume, a correlation with PAT could only be demonstrated for epithelioid PM [64].

Faccioli et al. assessed the significance of sarcopenia as a forecaster of short- and long-term results in subjects surgically managed for MPM [65]. In their study, they included subjects managed with a cytoreductive intent in a multimodality setting, having both pre- and post-operative CT scans without contrast available, and they excluded subjects who had not achieved complete macroscopic resection [65]. In total, 86 subjects [mean age: 66 (62–71 years), 76% males] participated in this study, and sarcopenia was evaluated by assessing the mean muscular density of the bilateral paravertebral muscles (T12 level) on pre-and post-operative CTs [65]. Concerning their results, it was demonstrated that sarcopenia was present pre-operatively in 57 (66%) subjects and post-operatively in 61 (74%). In addition, post-operative subjects with sarcopenia had a decreased 3-year overall survival (OS) in comparison with those who were non-sarcopenic (34.9% vs. 57.6% *p* = 0.03), while pre-operative sarcopenia was importantly related to an increased rate of post-operative adverse outcomes (65% vs. 41%, *p* = 0.04) [65]. They concluded that the assessment of sarcopenia utilizing a non-invasive manner would be of great importance to better pick subjects submitted to MPM operations [65].

## 4. Discussion

In this review article, we examined the possible interaction between PM and sarcopenia. It is already recorded that there is an interplay between sarcopenia and malignancies, and more specific, sarcopenia was significantly related to poorer prognosis across 12 types of cancer, among them lung, esophageal, gastric, hepatocellular, pancreatic, urothelial, head and neck, breast, colorectal, hematologic malignancies, and ovarian [66,67,68,69,70,71,72,73,74,75,76,77,78,79,80]. Additionally, in subjects diagnosed with cancer and cancer survivors, it has already been demonstrated that they have an accelerated decline in appendicular lean muscle mass and muscle mass loss in comparison with non-cancer subjects which might be related to impairment in physical function [66,67,68,81,82,83].

All these claims fueled our initial intention to investigate the existence of any potential interplay between sarcopenia and PM. Of course, there are certain limitations. It seems that the number of the existing studies is small and the data are scarce, and the follow-up periods of subjects included are mostly small for most of the studies. In addition, most of the studies seems to have enrolled a small number of participating subjects and might derive from a single medical and research center. As a result, it would be of great importance to conduct studies concerning larger number of participating subjects deriving from different study centers across the world and places with different epidemiology concerning these two medical entities. Moreover, the PM patients’ follow-up period could be larger than the follow-up period in the existing literature. Concerning the screening and diagnosis methods of sarcopenia in these subjects, it would be of significant importance to have more studies utilizing the DXA scan method which seems to be generally the most broadly utilized technique, as abovementioned.

An intriguing issue, could be the investigation of the potential interaction that might exist between chemotherapy regimens for PM and skeletal muscle mass and the effect of these regimens on sarcopenia. It is important to examine whether these regimens affect the skeletal muscle mass health and which would be the best drug and dosage to administrate in order not to deteriorate further a potential pre-sarcopenic state of a PM patient. In addition, it is already recorded that both low muscle mass and low muscle attenuation have been related to decreased chemotherapy tolerance in general [84]. As a result, this hypothesis should be further investigated in PM subjects.

It would be quite interesting if we could investigate the optimum nutritional supplementation and interventions among subjects with PM, which could have a positive impact on muscle mass and strength in sarcopenic subjects with PM, and also study the potential physical exercise interventions that we could implement on these subjects. It has already been demonstrated that nonpharmacological means to reduce sarcopenia during chemotherapy consists of resistance training and dietary counselling. Pharmacologic management might include omega-3 fatty acids, vitamin D replacement if depleted, testosterone and selective androgen receptor modulators (SARMS), and ghrelin [85].

It seems imperative to have a group of specialists and scientists, among them physical trainers, nutritionists and physicians, to collaborate in order to provide the optimum healthcare service in these patients. In addition, it would be quite significant if surgeons confronting PM subjects could include in their routine a pre-operative evaluation, the sarcopenia assessment using the existing screening tools. It is already well established that a variety of tools to screen sarcopenia can be utilized in everyday clinical practice. Among them, as it has already been mentioned, are the SARC-F questionnaire and hand grip strength dynamometry, which can be easily applied in these subjects [86,87,88,89,90,91,92,93]. These might provide the opportunity to identify pre-sarcopenic and sarcopenic PM subjects and try to ameliorate their skeletal muscle mass health before the procedure, avoiding potentially adverse post-operative conditions that might be associated with sarcopenia and low skeletal muscle mass.

## 5. Conclusions

To conclude, muscle loss in PM subjects was related to poorer survival and lower levels of activity. It seems that subjects with PM had increased rates of pre-sarcopenia and malnutrition, while pre-sarcopenia was related to worse activity levels, and malnutrition was related to worse QoL. Both sarcopenia and tumor volume were related to long-term mortality in surgically managed PM subjects, whilst sarcopenia was present both pre-operatively and post-operatively in these patients. In addition, post-operative sarcopenic subjects had a decreased 3-year OS than those without sarcopenia, while pre-operative sarcopenia was importantly associated with an augmented rate of post-operative adverse results. Nevertheless, more studies are imperative to authenticate these claims and crystallize this intriguing interplay. In addition, more investigations are needed to validate the potential prognostic profile of sarcopenia pre-operatively in order to avoid upcoming adverse outcomes.

## Figures and Tables

**Table 1:** Sarcopenia and pleural mesothelioma upcoming associations.

Authors/[Ref]	Study Type	Study Population	Main Results	Sarcopenia Evaluation
Jeffery et al. [62]	Cross-sectional analysis	61 MPM subjects, 79% male with median age 69 (62–74) years	54% were pre-sarcopenic and 38% were malnourished. The light activity period percent per day was lower in subjects with pre-sarcopenia in comparison with non-sarcopenic subjects (*p* = 0.008). Subjects with malnutrition had worse HRQoL than well-nourished subjects (*p* < 0.001).	ASM measured via DXA
Jeffery et al. [63]	Observational study	18 MPM subjects (89% men, mean age 68.9 ± 7.1 years)	In comparison with subjects with ASM maintenance (n = 9), fewer subjects with ASM loss (n = 9) survived ≥12 months from follow-up (*p* = 0.002). Subjects with ASM loss had augmented sedentary time (*p* = 0.028) and lowered light activity (*p* = 0.028) and step count (*p* = 0.008). Activity levels did not alter in subjects with ASM maintenance (*p* > 0.05), while both energy and protein intake did not demonstrate any alteration in either group (*p* > 0.05).	DXA
Verhoek et al. [64]	Retrospective study	278 PM-subjects (252 male, 70.2 ± 9 years)	Mean progression-free survival was 18.6 ± 12.2 months. Mean survival period was 23.3 ± 24 months. Progression related to COPD (*p* < 0.001), type of surgery (*p* = 0.026), tumor stage (*p* = 0.001). Three-year mortality related to higher subject age (*p* = 0.005), increased tumor stage (*p* = 0.015), presence of COPD (*p* < 0.001), increased tumor volume (*p* < 0.001). Sarcopenic subjects had increased three-year mortality (*p* = 0.002). Even though there was an inverse correlation of progression-free survival and mortality with tumor volume (r = 0.281, *p* = 0.001 and r = −0.240, *p* < 0.001, respectively), a correlation with PAT was only demonstrated for epithelioid PM (*p* = 0.040)	CT-based parameters evaluated at TH5 level, excluding fatty infiltration based on CT attenuation
Faccioli et al. [65]	Single-center retrospective study	86 subjects surgically treated for MPM [mean age: 66 (62–71 years), 76% males]	Sarcopenia pre-operatively present in 57 (66%) subjects and post-operatively in 61 (74%). Post-operative sarcopenic subjects had decreased 3-year OS than non-sarcopenic (*p* = 0.03). Pre-operative sarcopenia importantly related to increased frequency of post-operative adverse outcomes (*p* = 0.04)	Mean muscular density of the bilateral paravertebral muscles (T12 level) on pre- and post-operative CTs

Abbreviations: MPM: malignant pleural mesothelioma; *p*: *p*-value; HRQoL: health-related quality of life; ASM: appendicular skeletal muscle mass; DXA: dual-energy X-ray absorptiometry; PM: pleural mesothelioma; COPD: chronic obstructive pulmonary disease; PAT: precardial adipose tissue; CT: computed tomography; TH5: fifth thoracic vertebra; OS: overall survival; T12: 12 thoracic vertebrae.

## Data Availability

The data presented in this study are available upon request from the corresponding author.
